# Microglia Increase Inflammatory Responses in iPSC-Derived Human BrainSpheres

**DOI:** 10.3389/fmicb.2018.02766

**Published:** 2018-12-04

**Authors:** Celina Monteiro Abreu, Lucio Gama, Susanne Krasemann, Megan Chesnut, Shelly Odwin-Dacosta, Helena T. Hogberg, Thomas Hartung, David Pamies

**Affiliations:** ^1^Department of Molecular and Comparative Pathobiology, Johns Hopkins School of Medicine, Baltimore, MD, United States; ^2^Vaccine Research Center, National Institute of Allergy and Infectious Diseases, NIH, Bethesda, MD, United States; ^3^Institute of Neuropathology, University Medical Center Hamburg-Eppendorf, Hamburg, Germany; ^4^Center for Alternatives to Animal Testing, Johns Hopkins Bloomberg School of Public Health, Baltimore, MD, United States; ^5^CAAT-Europe, University of Konstanz, Konstanz, Germany

**Keywords:** zika virus, organoids, microglial cells, *in vitro* culture, BrainSpheres

## Abstract

Human induced pluripotent stem cells (iPSCs), together with 21st century cell culture methods, have the potential to better model human physiology with applications in toxicology, disease modeling, and the study of host-pathogen interactions. Several models of the human brain have been developed recently, demonstrating cell-cell interactions of multiple cell types with physiologically relevant 3D structures. Most current models, however, lack the ability to represent the inflammatory response in the brain because they do not include a microglial cell population. Microglia, the resident immunocompetent phagocytes in the central nervous system (CNS), are not only important in the inflammatory response and pathogenesis; they also function in normal brain development, strengthen neuronal connections through synaptic pruning, and are involved in oligodendrocyte and neuronal survival. Here, we have successfully introduced a population of human microglia into 3D human iPSC-derived brain spheres (BrainSpheres, BS) through co-culturing cells of the Immortalized Human Microglia – SV40 cell line with the BS model (μBS). We detected an inflammatory response to lipopolysaccharides (LPS) and flavivirus infection, which was only elicited in the model when microglial cells were present. A concentration of 20 ng/mL of LPS increased gene expression of the inflammatory cytokines interleukin-6 (*IL-6*), *IL-10*, and *IL-1β*, with maximum expression at 6–12 h post-exposure. Increased expression of the *IL-6, IL-1β*, tumor necrosis factor alpha (*TNF-α*), and chemokine (C-C motif) ligand 2 (*CCL2*) genes was observed in μBS following infection with Zika and Dengue Virus, suggesting a stronger inflammatory response in the model when microglia were present than when only astrocyte, oligodendrocyte, and neuronal populations were represented. Microglia innately develop within cerebral organoids (Nature communications)^1^, our findings suggest that the μBS model is more physiologically relevant and has potential applications in infectious disease and host-pathogen interactions research.

## Introduction

Microphysiological systems and organotypic cell culture models are increasingly being used to model human physiology *in vitro* because these models better mimic the *in vivo* situation compared to traditional monolayer cell culture systems (Marx et al., 2016; Pamies and Hartung, 2017; Smirnova et al., 2018). In addition, such models allow for the mechanistic understanding of biological processes that would be challenging to study *in vivo*. In particular, the human brain is a highly complex structure that cannot easily be interrogated *in vivo* and has proven difficult to model *in vitro*. Recently, the first *in vitro* human organotypic models of the brain have been developed, including 3D structures and heterogeneous cell populations. Many of these models seek to represent key events in human brain development and cell-cell interactions between various cell types (Kadoshima et al., 2013; Lancaster et al., 2013; Pasca et al., 2015; Pamies et al., 2017; Sandstrom et al., 2017). The basis for the majority of these models is a neural progenitor cell (NPC) population; however, microglia, arise from erythromyeloid precursor cells in the embryonic yolk sac, unlike the other cell types in the brain which arise from the neuroectoderm (Alliot et al., 1999; Ginhoux et al., 2013; Kierdorf et al., 2013; Sousa et al., 2017). Thus, because microglia cannot be derived from NPCs, many of the current *in vitro* models of the human brain do not include a microglial cell population.

Microglia are the resident mononuclear phagocytic cells in the central nervous system (CNS) (Ginhoux et al., 2013). These cells are both neuroprotective and immunocompetent, as they play critical roles in brain development, strengthen neuronal connections through synaptic pruning, are involved in neuronal maintenance and support, and are responsible for the inflammatory response in the brain following an injury or pathogenic infection (Kettenmann et al., 2011; Paolicelli et al., 2011; Michell-Robinson et al., 2015; Thompson and Tsirka, 2017). In response to local alterations, lesions, or pathogen invasion, microglia become active. Microglial activation involves changes in morphology, the release of multiple substances such as proinflammatory cytokines, chemokines, and reactive oxygen species, migration to affected areas, proliferation, and phagocytosis of cell debris (Kettenmann et al., 2011; Boche et al., 2013; Thompson and Tsirka, 2017). This process is also a prominent feature of inflammation in neurodegenerative diseases such as Alzheimer’s disease, Parkinson’s disease, multiple sclerosis, and infectious processes (Wang et al., 2015; Thompson and Tsirka, 2017).

Various models have been developed to study neurological disorders and lipopolysaccharide (LPS) has been widely used as an inducer of neuroinflammation and neurotoxicity as it has been shown to act as a potent stimulator of microglia (Gao et al., 2002; Hu et al., 2012; Olajide et al., 2013; Kempuraj et al., 2017). Additionally, inflammation related neurodegeneration induced by LPS is a common approach to study mechanisms of cellular neuroimmunology (Hu et al., 2012). Neurotropic virus infections are major pathogens in the CNS and usually cause inflammation (Ludlow et al., 2016). The *Flavivirus* genus constitutes some of the most serious human pathogens, including Japanese encephalitis (JEV), dengue (DENV), zika (ZIKV), and yellow fever (YF), all of which are capable of invading the central and peripheral nervous system and are regarded as neurotropic viruses (Neal, 2014). The mechanisms by which flaviviruses alter the immune and the CNS have only recently been examined, but remain largely unclear (Daep et al., 2014). The NPC model has been used in ZIKV studies to understand mechanisms that govern neuropathogenesis and immunopathogenesis in CNS infection (Dang et al., 2016; Garcez et al., 2016; Tang et al., 2016; Qian et al., 2017), but these models do not contain microglia.

We previously developed a 3D brain model (BrainSpheres, BS) derived from human-induced pluripotent stem cells (iPSCs) and demonstrated that this model is reproducible with respect to size and shape and is comprised of populations of neurons, astrocytes, and oligodendrocytes (Pamies et al., 2017). In addition, the model has been applied to the study of developmental neurotoxicity (Pamies et al., 2018). In this study, we introduced a population of human microglia into the BS model and compared the inflammatory response of BS without microglia to BS co-cultured with microglia (μBS) after treatment with LPS, ZIKV, or DENV. In addition, we quantified cell death following ZIKV infection because there is evidence that ZIKV infection can impair brain growth via cell death of developing neurons.

## Materials and Methods

### Induced Pluripotent Stem Cell Generation

Fibroblasts (American Type Culture Collection (ATCC), Manassas, VA, United States, CCD-1079Sk ATCC^®^ CRL-2097^TM^) were used to generate iPSCs as previously described (Wen et al., 2014; Pamies et al., 2017). iPSCs (passage ≤20) were cultured on irradiated MEFs in human embryonic stem cell (hESC) medium comprised of D-MEM/F12 (Invitrogen, Carlsbad, CA, United States) 20% Knockout Serum Replacement (KSR, Invitrogen, Carlsbad, CA, United States), 2 mM L-glutamine (Invitrogen, Carlsbad, CA, United States), 100 μM MEM NEAA (Invitrogen, Carlsbad, CA, United States), 100 μM β-mercaptoethanol (Invitrogen, Carlsbad, CA, United States), and 10 ng/mL human basic FGF (bFGF, PeproTech, Rocky Hill, NJ, United States). Cell culture medium was changed daily. iPSCs were passaged using collagenase (Invitrogen, Carlsbad, CA, United States, 1 mg/mL in D-MEM/F12 for 1 h at 37°C).

### Neural Progenitor Cell Production

NPC were generated following the previously published protocol (Wen et al., 2014). NPCs were cultured with StemPro^®^ NSC SFM media (Life Technologies, Carlsbad, CA, United States) in 175 cm^2^ flasks (Thermo Scientific, Waltham, MA, United States, Nunc^TM^ Filter Cap EasYFlask^TM^) coated with Poly-L-ornithine hydrobromide (Sigma-Aldrich, St. Louis, MO, United States, P3655) and laminin (Sigma-Aldrich, L2020, from Engelbreth-Holm-Swarm murine sarcoma basement membrane). Half of the media was changed daily. Cultures were maintained at 37°C with 5% CO_2_. NPCs were passaged by mechanical detachment with a cell scraper (Sarstedt, NC, United States, 2-position, Blade 25, 83.1830).

### BrainSpheres Differentiation

The production of BS was published previously (Pamies et al., 2017). Briefly, to produce BS, NPCs were detached mechanically with a cell scraper (Sarstedt, 2-position, Blade 25, 83.1830), re-pipetted for disaggregation, and counted using the Countess Automated Cell Counter (Invitrogen, Carlsbad, CA, United States). 2 × 10^6^ cells per well were plated in non-treated Falcon^TM^ Polystyrene 6-well plates (Corning, Corning, NY, United States). Cells were grown in differentiation medium [Neurobasal^®^ electro Medium (Gibco, Gaithersburg, MD, United States)] supplemented with 2% B-27^®^ Electrophysiology (Gibco), 1% GlutaMAX (Gibco), 0.01 μg/mL human recombinant GDNF (Gemini, Woodland, CA, United States), and 0.01 μg/mL human recombinant BDNF (Gemini). Cultures were maintained at 37°C in an atmosphere of 5% CO_2_ under constant gyratory shaking (88 rpm) for 7 weeks. Differentiation medium was changed every 2 days.

### Microglia Culture

Immortalized Human Microglia – SV40 (Applied Biological Materials Inc., Richmond, BC, Canada) were grown in non-treated 75 cm^2^ flasks (Thermo Scientific, Waltham, MA, United States, Nunc^TM^ Filter Cap EasYFlask^TM^). Cells were expanded using Prigrow III medium (Applied Biological Materials Inc.) supplemented with 10% Fetal Bovine Serum (Gibco, Gaithersburg, MD, United States, Certified, Heat Inactivated, United States Origin) and 1% Penicillin-Streptomycin (Gibco, 10,000 U/mL). Microglia were passaged by mechanical detachment using a cell scraper (Sarstedt, 2-position, Blade 25, 83.1830). Cultures were maintained at 37°C in an atmosphere of 5% CO_2_ immortalized Human Microglia – SV40 cell line was declared mycoplasma free by a mycoplasma testing using a PCR based MycoDtect kit from Greiner Bio-One (Genetic Resource Core Facility is a core resource of the Johns Hopkins School of Medicine, Institute of Genetic Medicine).

### Formation of BS With Microglia (μBS)

The density of microglia added to the aggregates was studied previously and the optimal number of cells was chosen to avoid the formation of microglia-only spheres and to allow enough microglia attachment to aggregates. BS were differentiated for 7 weeks at which time, 3 × 10^5^ microglia/well were added to the BS suspension. Upon addition of microglia, plates were kept in static conditions in the incubator at 37°C with 5% CO_2_ for 24 h, with manual shaking of the plates every 6 h. This allowed for the attachment of the microglia to the surface of the BS while avoiding BS agglomeration. Microglia which did not attach to BS during this time attached to the bottom of the well. We then carefully removed aggregates from the well and transferred them to a new plate. μBS aggregates were then washed twice with PBS, and new differentiation media was added. After the incorporation of microglia, μBS were maintained in culture for one more week.

### Histology and Immunohistochemistry

BS with and without incorporated microglia were fixed in 4% formalin/PBS. After fixation, spheres were carefully embedded into low-melting agarose and the solidified mixture was processed into paraffin blocks using an ASP300S dehydration machine (Leica, Wetzlar, Germany) and an EG1160 tissue embedding system (Leica, Wetzlar, Germany). Sections (2 μm) were stained with hematoxylin-eosin (H&E) or processed for immunohistochemistry as follows: After dewaxing and inactivation of endogenous peroxidases (PBS/3% hydrogen peroxide), antibody specific antigen retrieval was performed using the Ventana Benchmark XT machine (Ventana, Tucson, AZ, United States). Sections were blocked (PBS/10% FCS) and afterward incubated with the primary antibodies TMEM119 (1:100; Sigma), Mertk (1:100; R&D), Axl (1:100; LSBio), CD11b (1:2,000; Abcam), and P2ry12 (1:100; Sigma). For double staining of microglia and neurons, microglia were detected with TMEM119 and visualized with DAB in brown, whereas neurons were stained with NeuN (1:50; Millipore) and incubated with anti-mouse AP-coupled secondary antibody and developed in red. Bound primary antibodies were detected with secondary antibodies using Histofine Simple Stain MAX PO immune-enzyme polymer (Nichirei Biosciences) and stained with 3,3^′^-Diaminobenzidine (DAB) substrate using the ultraView Universal DAB Detection Kit (Ventana). Tissues were counterstained with hematoxylin. Representative images were taken with a Leica DMD108 digital microscope.

### Virus Propagation and Titering

Vero (ATCC, Manassas, VA, United States, ATCC^®^ CCL81^TM^) and BHK-21 (ATCC^®^, C-13 ATCC CCL-10) cell lines were cultured in D-MEM (Gibco) containing 10% Fetal Bovine Serum (Gibco, Certified, Heat Inactivated, United States Origin), 2 mM L-glutamine (Invitrogen), Penicillin-Streptomycin (Gibco 10,000 U/mL), and 10 mM Hepes buffer (Gibco) in an incubator at 37°C with 5% CO_2_. Vero and BHK-21 cells were passaged 3 times a week. To passage Vero and BHK-21 cells, the cells were incubated with 0.05% trypsin (Gibco) in 1× PBS (Gibco) at 37°C with 5% CO_2_ for 5 min, resuspended in 10 mL D-MEM, and centrifuged at 1200 rpm for 8 min. The supernatant is then removed and the cells were resuspended in fresh media and transferred to a new flask.

Two strains of ZIKV (ATCC, VR-1838^TM^, Uganda 1947, ZIKV-UG, and Brazil 2015, ZIKV-BR) were propagated in Vero cells and Dengue Virus type 1 (ATCC, VR-1586^TM^, DENV-1) was propagated in BHK-21 cells. ZIKV-BR was isolated from a febrile non-pregnant Brazilian woman with a rash in Paraiba, Brazil in 2015 and kindly provided by Professor Pedro Vasconcelos from the Instituto Evandro Chagas, Belém, State of Pará, Brazil (Pompon et al., 2017). ZIKV-UG was isolated in 1947 from a rhesus macaque exposed to mosquitos in Uganda. The Viral stocks were prepared by infecting Vero or BHK-21 cells at 80–90% confluency in 25 cm^2^ flasks with 2 mL of virus dilution in OptiPro^TM^ SFM media (Gibco) at a multiplicity of 0.1. The cells were incubated for 6 h at 37°C with 5% CO_2_, then the supernatant was removed and the cell lines were washed two times with 1× PBS and replaced with 10 mL of D-MEM. The infected cells were further incubated for 5 days, and then the supernatants were collected and transferred to a 75 cm^2^ flask with respective cell lines at 90% confluence. The ZIKV-UG, ZIKV-BR, and DENV-1 were collected after 7 days post-infection (p.i.), clarified by centrifugation at 500 ×*g* for 10 min, and filtered through a 0.22-μm membrane. All viral stocks were stored in 1 mL aliquots at -80°C. Virus titers used in the assays were determined by double-overlay plaque assay of Vero and BHK-21 cells to ZIKV and DENV, respectively, as previously described (Baer and Kehn-Hall, 2014).

### Lipopolysaccharide Treatment

After 8 weeks of differentiation, 2 μl from a 10 μg/mL stock of LPS were added to 2 mL media containing ether the microglia, BS or μBS, obtaining a final concentration of 20 ng/mL. Samples were collected 3, 6, 12, and 24 h after the exposure. Time 0 refers to samples before LPS exposure. Samples were collected for qPCRs.

### Viral Infection

Immortalized Human Microglia – SV40 were plated in 24-well plates at a density of 2.5 × 10^5^ cells per well. The BS and μBS or microglia from one well were transferred to a 24-well-plate (and divided into triplicates) before infecting with ZIKV-UG, ZIKV-BR, and DENV-1. A MOI of 0.1 for 6 h at 37°C with 5% CO_2_ was used. Three wells of non-infected BS, μBS, and microglia were used as control (MOCK). The cells were washed 3 times with 500 μl PBS, and then 250 μl of supernatant were collected for each condition as time zero of infection. The PBS was totally removed and BS, μBS or microglia were re-suspended in fresh complete media. BS and μBS were then transferred back to a 6-well plate containing media. The plates containing the infected and uninfected cells were incubated at 37°C with 5% CO_2_ for 24 to 72 h. The supernatant was collected at different times points 24, 48, and 72 h post infection (p.i.) to check viral load. The cells were collected at 48 and 72 h p.i. to analyze gene expression, Annexin-V, and cell cycle (3 technical replicates). One well of BS, μBS and microglia were used for confocal microscope analysis. All work with infectious ZIKV was performed in an approved BSL-3 facility.

### Analysis of Cell Viability and Cytotoxicity Using Flow Cytometry

Apoptosis in microglia cells, BS, and μBS was assayed using the Muse Annexin V and Dead Cell kit (Millipore, Billerica, MA, United States) according to the user guide and the manufacturer’s instructions. After 72 h incubation at 37°C in 5% CO_2_, microglia cells for all conditions were harvested through mechanical detachment with a cell scraper (Sarstedt, Blade 25, 83.1830) in 500 μl of the PBS, and centrifuged for 5 min at 2000 RPM. Two aggregates of BS and μBS were collected using a 1 mL micropipette. The pellet was resuspended in 100 μL of the PBS with 1% FBS, mixed with 100 μL of the Muse Annexin V and Dead Cell Reagent at room temperature. Tubes were mixed using a vortex for 10 s. Samples were incubated for 20 min at room temperature and protected from light. The percentages of apoptotic cells were analyzed by flow cytometry using Muse Cell Analyzer (Millipore, United States) system and values were expressed as mean of apoptotic cells relative to mock with error bars representing the SD. All values are expressed as mean ± SD (*n* = 3). Statistical significance (defined as *P*-value < 0.05) was evaluated using multiple *t*-test to compare control and infected or treated cells (Graph Prism 7 Software, San Diego, CA, United States).

### Flow Cytometry Analysis of Cell Cycle

Microglia cells, BS and μBS (infected and uninfected) were cultivated in 6-well plates and incubated for 48 h. Microglia cells for all conditions were harvested through mechanical detachment with a cell scraper (Sarstedt, Blade 25, 83.1830) in 500 μl of the PBS, and centrifuged for 5 min at 2000 RPM. Two aggregates of BS and μBS were collected using a 1 mL micropipette. Obtained pellets were fixed with 70% ethanol. The cells were kept in -20°C overnight. After ethanol removal, cells were suspended in 250 μL PBS and centrifuged for 5 min at 2000 RPM. Cell pellets were suspended in 200 μL of Muse Cell Cycle Reagent and were incubated for 30 min at room temperature and protected from light. The cell suspension was transferred to a 1.5 mL microcentrifuge tube prior to analysis on Muse Cell Analyzer. Cell cycle was assessed by fluorescence-activated cell analysis using a Muse Cell Analyzer (Merck, Millipore, United States). All values are expressed as mean ± SEM (*n* = 3). Statistical significance (defined as *P*-value < 0.05) was evaluated using multiple *t*-test to compare control and infected or treated cells with LPS (Graph Prism 7 Software).

### Viral RNA Extraction

Viral RNA for the real-time RT-PCR (qPCR) assays was purified from the culture supernatant of the uninfected and infected microglia, BS and μBS. The viral RNA was extracted from 240 μl of culture supernatant by using the QIAamp MinElute virus spin kit (Qiagen, Valencia, CA, United States) according to the manufacturer’s instructions, with the exception of the elution volume, which was 65 μl.

### DENV-1 and ZIKV qPCR

RNA isolated from the supernatant after DENV-1 and ZIKV infections were used in qPCR independently. The MOCK samples were used as negative controls in both qPCRs. The QuantiTect One-Step RT-PCR kit (Qiagen, Hilden, Germany) was used with a 25 μl reaction mixture under the following conditions: 5 μl of kit master mixture (including Taq polymerase and RT enzyme), 1.25 μl of 10 μM of each primer, 0.5 μl of 10 μM of probe, 7 μl of RNA-free-water (Mol Bio grade, Hamburg, Germany), and 10 μl of the extracted sample. Each amplification run contained negative control (NC) of each experiment (MOCK); non-template control (NTC), and positive control (PC). The non-template control consisted of blank reagent and water. For the positive control, nucleic acid extracted from each virus stock were used after dilution 1:1000 to avoid cross-contamination. The protocol used to ZIKV and DENV-1 qPCR was the same conditions except primers and probes (Table 1) and cycling thermal. The DENV-1 qPCR was done as described previously (Johnson et al., 2005) with some modification; a single cycle of reverse transcription for 30 min at 50°C, 15 min at 95°C for reverse transcriptase (RT) inactivation, and DNA polymerase activation followed by 45 amplification cycles of 15 s at 95°C and 1 min 55°C (annealing-extension step). The following thermal profile was used to ZIKV qPCR a single cycle of reverse transcription for 30 min at 50°C, 15 min at 95°C for RT inactivation, and DNA polymerase activation followed by 40 amplification cycles of 15 s at 95°C and 1 min 60°C (annealing-extension step). For RNA standards, RNA was isolated from purified, titered stock of ZIKV-UG or DENV-1. RNA yield was quantified by spectrometry and the data was used to calculate genomes/μl. ZIKV-UG and DENV-1 RNA was serially diluted 1:10 in 8 points of dilution for standard curve (10^7^ to 10^1^). The standard curve was amplified in duplicate using primers and conditions described above for each specific qPCR. The number of infectious viral RNA transcripts detected was calculated by generating a standard curve from 10-fold dilutions of RNA isolated.

**Table 1:** Oligonucleotide primers and fluorogenic probes used in DENV-1 and ZIKV virus qPCR assay.

Virus detection	Sequence 5^′^–3^′^	Genome position	Fluorophore
DENV-1 reverse primer	CAAAAGGAAGTCGTGCAATA	8973–8993	-
DENV-1 reverse primer	CTGAGTGAATTCTCTCTACTGAACC	9084—9109	-
DENV-1 probe	CATGTGGTTGGGAGCACGC	8998—9017	FAM/BHQ-1
ZIKV forward primer	AARTACACATACCARAACAAAGTGGT	9271–9297	-
ZIKV reverse primer	TCCRCTCC CYCTYTGGTCTTG	9352–9373	-
ZIKV probe	CTYAGACCAGCTGAAR	9304–9320	FAM/BHQ-1


### Analysis of Confocal Microscopy

After 72 h exposure BS and μBS were collected and fixed with PFA (4%) for 1 h at room temperature. Subsequently, BS, and μBS were washed twice with PBS (1×) and incubated with blocking solution (10% goat serum, 1% BSA, 0.15% saponin in PBS) at 4°C for 1 h. The BS were washed with washing solution (1% BSA, 0.15% saponin in PBS), and incubated overnight at 4°C with primary antibodies: Zika NS1 (OWL, 55788, 1:200, ZIKV) anti-NF200 (Sigma Aldrich, N4142, 1:200, neurofilament for all neurons), and IBA1 (WAKO, Richmond, VA, United States, 019-19741, 1:200, microglia) diluted in blocking solution. The next day the BS and μBS were washed twice with washing solution and incubated with the secondary antibody 568-Alexa anti-rabbit and 488-Alexa anti-mouse both 1:200 diluted in blocking solution for 24 h at 4°C and protected from light. Thereafter, BS and μBS were washed twice again with washing solution. Nuclei were stained with Hoechst 33342, diluted 1: 10,000 in PBS for 1 h. The BS were transferred to microscope glass slides and mounted with “Immuno mount” and imaged with a confocal microscope Zeiss LSM 510 Confocal III (Zeiss) with a 20× objective.

### Cellular RNA Isolation and qPCR

Total RNA was extracted from microglia, BS and μBS 72 h post infection (p.i.) using RNAeasy Mini Kit (Qiagen, Hilden, Germany). RNA quantity and purity was determined using a NanoDrop 2000c (Thermo Scientific). 1 μg of RNA was reverse-transcribed using the MLV Promega RT (Promega) according to the manufacturer’s recommendations. The expression of genes was evaluated using specific TaqMan^®^ Gene Expression Assays (Life Technologies). Table 2 shows a summary of the assayed genes. qPCR was performed using a 7500 Fast Real Time system machine (Applied Biosystems). Fold changes were calculated using the 2 (-ΔΔCt) method (Livak and Schmittgen, 2001). β-actin and 18s were used as housekeeping genes for mRNA. Data are presented as mean ± SD, normalized to housekeeping genes and MOCK.

**Table 2:** List of TaqMan assays used for qPCR analysis.

*CCL2*	Chemokine (C-C motif) ligand 2	Hs00234140_m1
*TNF-*α	Tumor necrosis factor alpha	Hs01113624_g1
*IL10*	Interleukin 10	Hs00961622_m1
*IL6*	Interleukin 6	Hs00985639_m1
*IL1b*	Interleukin 1 beta	Hs01555410_m1
*ACTB*	Beta-actin	Hs01060665_g1


## Results

### Incorporation of SV40-Immortalized Human Microglial Cells Into a 3D BS Model

**FIGURE 1:**
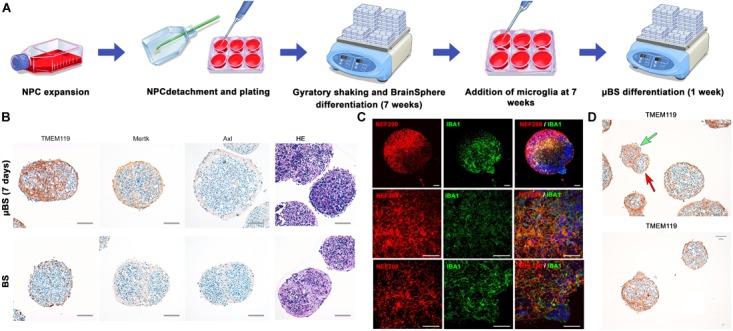
Generation of microglia-containing BS (μBS). Immortalized Human Microglia – SV40 were incorporated to 7 week-differentiated BS by gravity as described in materials and methods. **(A)** Diagram of μBS generation procedure. **(B)** Comparison between μBS and BS using different microglia markers (TMEM119, Mertk, Axl) and hematoxylin/eosin (HE). **(C)** Confocal images of immunohistochemistry for the microglia marker IBA1 (green), the neuronal marker NF200 (red), and nuclear staining (Hoechst 33342, blue) in μBS, 48 h after microglia incorporation. **(D)** Details of microglia aggregations in μBS. Green arrow indicates microglia cluster and red arrow indicates the BS. Bars represent 100 μm **(B)**, 50 μm (**C**, upper panel), 20 μm (**C**, all other panels), and 50 μm **(D)**.

Microglia cells were added to the BS at 7 weeks of differentiation (Figure 1A) and were able to attach to the surface of the BS. However, as the microglia cell line used for this study is immortalized and proliferation takes place, we were only able to maintain μBS (BS with microglia) for 1 week after incorporation for our experiments. Immunohistochemistry of μBS demonstrated they were positive for microglia markers such as TMEM19, Mertk, Axl (Figure 1B), and IBA1 (Figure 1C). Microglia grew on the surface of the μBS, forming a microglia protuberance on the sphere (Figures 1C,D). These formations appear in 50% of the aggregates, seemingly due to the co-culture technique.

### Presence of Microglia in BS Alters Gene Expression of Cytokines in Response to Inflammatory Stimuli

**FIGURE 2:**
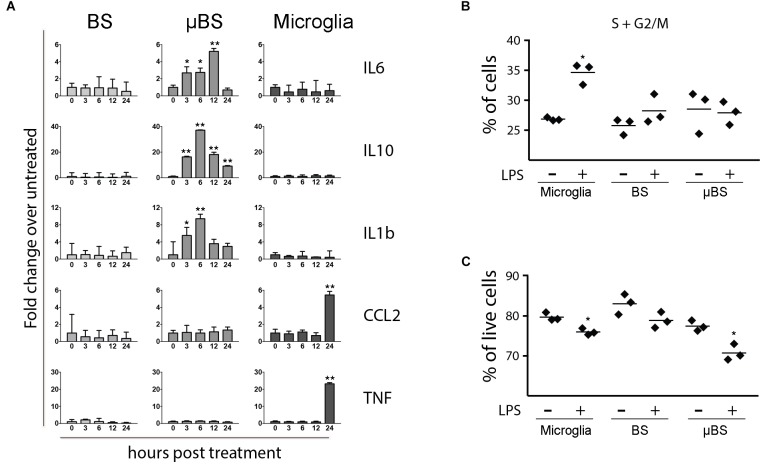
Differences between BS, μBS, and microglia after LPS treatment. The microglia, BS, μBS, were exposed to 20 ng/mL LPS up to 24 h. **(A)** shows gene expression of IL6, IL10, IL1b, CCL2, and TNFa after LPS treatment on the different models (BS, μBS, and microglia cells). Data are shown as fold change of treated versus untreated up to an hour post treatment (3, 6, 12, and 24 h after treatment). **(B)** show changes in cell cycle in percentage of the cells in G2 plus S phase in microglia cells, BS and μBS treated or non-treated with LPS. Each dot symbol represent a replicate sample (n+3). **(C)** shows Annexin V Apoptosis Muse assay. Results show % live cells microglia, BS, ad μBS cells treated (+) or not treated (–) with LPS. Each symbol represent a replicate (*n* = 3). Statistical analysis in A was performed using Dunnett test on ΔΔCt. The asterisks symbols represents ^∗^*P* < 0.05 and ^∗∗^*P* < 0.01 (two independent experiments and three biological replicates on each experiment).

Once microglial cells were successfully incorporated into BS, we evaluated whether the μBS model would then respond to inflammatory stimuli in a distinct manner to BS. Microglial culture, BS, and μBS were exposed to 20 ng/mL LPS, which is a potent activator of inflammatory pathways in myeloid cells (Fujihara et al., 2003). Cells were collected at 0, 3, 6, 12, and 24 h post-treatment (p.t.) and levels of *CCL2, TNF-α, IL-1β, IL-6,* and *IL-10* RNA were quantitated by qPCR. In the absence of microglia, BS responded poorly to LPS (Figure 2A). Conversely, cultures containing solely microglial cells responded to LPS by upregulating the expression of *CCL2* and *TNF-α* 24 h p.t. No significant changes in the levels of *IL-6, IL-1β,* and *IL-10* were observed. The mixing of both populations into μBSs, however, led to distinct responses against LPS, specifically for *IL-1β, IL-6*, and *IL-10*, which were all upregulated in the first 12 h p.t. and downregulated 24 h p.t. The increased RNA expression of *CCL2* and *TNF-α* in sole microglia cultures was not observed in μBS cultures, indicating that the presence of other cell types in the BS indirectly (through secreted factors) or directly (by cell-to-cell interactions) altered the LPS-response, which was observed in myeloid cells. This suggests crosstalk between microglia and other cell populations in the μBS model. After LPS treatment, microglia showed an increase in dividing cells, characterized by a higher percentage of cells in S or G2/M phases when compared to non-treated microglia (Figure 2B). In addition, cell viability decreased after LPS treatment in microglia and in μBS, indicating that microglia may play an important role in viral neurotoxicity (Figure 2C).

### The μBS Support Replication of Dengue 1 (DENV-1) and Zika Viruses (ZIKV)

**FIGURE 3:**
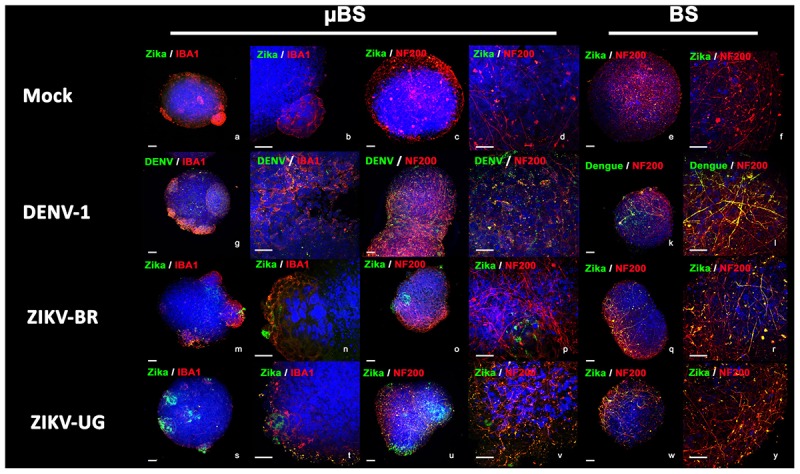
Virus infection in BrainSpheres. Figure shows virus infection by immunohistochemistry in BrainSpheres with (μBS) and without microglia (BS). Green represents the Flavivirus marker for ZIKV (*NS1*) and DENV-1, red represents microglia markers *IBA1* and *NF200*. Bars represent 50 μm (lower magnification) and 10 μm (higher magnification).

**FIGURE 4:**
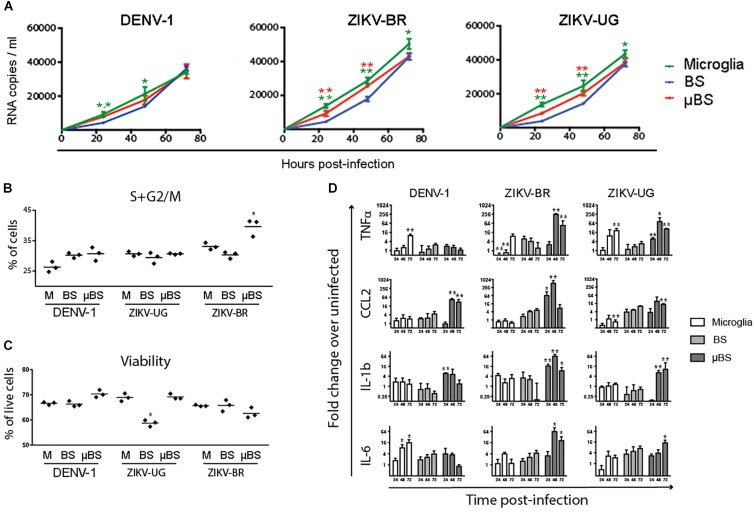
Effects of Flavivirus infection on 3D human iPSC-derived brain spheres (BrainSpheres, BS) without or with microglia cells (μBS). The microglia cells, BS, and μBS were infected with Dengue, ZIKV-BR, and ZIKV-UG using MOI equal 0.1. **(A)** shows the growth kinetics of flaviviruses over time for the three models. The viral load (RNA copies/mL) are shown with standard deviation. **(B)** Changes in cell cycle shown as percentage of cells in G2/S phase in BS, μBS, and microglia treated or non-treated with the different viruses. **(C)** Annexin V Apoptosis Muse assay results. Results represent % live cells in BS, μBS, and microglia samples after the different flavivirus treatments. **(D)** Gene expression relative quantification for *TNFα, CCL2, IL-1b*, and *IL-6* over the time. Statistical analysis was performed using the Dunnett’s Test (^∗^mean equal *p* < 0.05 and ^∗∗^*p* < 0.01) for two independent experiments, three biological replicates on each experiment.

Organoids have been previously used as models for virus pathogenesis (Gabriel et al., 2017; Salick et al., 2017; Watanabe et al., 2017). To evaluate whether the incorporation of microglial cells into BS would interfere with viral replication, microglia, BS, and μBS were infected with DENV-1 and two distinct strains of ZIKV (ZIKV-UG and ZIKV-BR). Figure 3 represents immunohistochemistry on BS and μBS for control (mock) and treated samples (ZIKV-BR, ZIKV-UG, and DENV). Mock samples did not show any positive staining for ZIKV (Figure 3) or DENV (data no shown). Cultured cells and aggregates were exposed to the three pathogens and viral growth in the supernatants was measured every 24 h for 3 days (Figure 4A). Microglia and μBS showed higher replication efficiency in the first 48 h showing a significant difference after 72 h in microglia infected with ZIKV-UG and ZIKV-BR. However, DENV-1 did not show significant changes between models after 72 h (Figure 4A). Viral infection was confirmed by immune staining (Figure 3). Both BS and μBS were found to be infected by ZIKV-BR, ZIKV-UG, and DENV which upon qualitatively analyzing the images, showed higher virus accumulation in microglia cells than in the other cell types (Figure 3). This together with our replication results (Figure 4A) could indicate that these flavivirus prefer to infect microglia or that microglia internalize more viral particles via phagocytosis.

DENV-1 infection led to a slight increase in dividing cells in BS and μBS, characterized by a higher percentage of cells in S or G2/M phases when compared to sole microglia (Figure 4B). However, DENV-1 was slightly more cytotoxic to microglia and BS than μBS (Figure 4C), indicating a possible protective role for microglial cells in the 3D model. Similar results were observed against ZIKV-UG, which appeared to be more cytotoxic to BS (Figure 4C), despite no changes in the cell cycle among all conditions (Figure 4B). This could be due to the protective capability of microglia and their role in internalizing viral particles. Interestingly, ZIKV-BR infection in μBS caused a significant increase in dividing cells concomitantly to a slight decrease in viability (not statistically significant), suggesting that microglial cells contribute to higher cytotoxicity in response to a more aggressive ZIKV strain.

### The Presence of Microglia Alters the Cytokine Gene Expression in Response to Flavivirus Infection

As part of the innate immune response, microglial cells respond to viral infection by secreting inflammatory cytokines. To determine whether the addition of microglial cells to BS modulates the immune response against flavivirus infection, the levels of intracellular *TNF-α, CCL2, IL-1b*, and *IL-6* mRNA were measured in sole microglia, BS, and μBS 72 h after exposure to DENV-1, ZIKV-UG, and ZIKV-BR. After DENV-1 infection, cytokine expression was observed mostly in microglia and μBS, but not BS. Interestingly, the secretion pattern varied between the analytes (Figure 4D). While *TNF-α* and *IL-6* production increased in sole microglia post-infection, *CCL2* and *IL-1b* were upregulated only in μBS (Figure 4D). The presence of microglia cells in μBS also led to an increase in ZIKV-induced cytokine production when compared to sole microglia and BS. Moreover, cytokine levels in μBS were significantly higher in response to ZIKV-BR than those observed with the Uganda strain.

## Discussion

3D brain models have been increasingly used in the last years, primarily in ZIKV studies (Qian et al., 2016, 2017; Wells et al., 2016; Salick et al., 2017; Watanabe et al., 2017; Zhou et al., 2017). These studies, however, have mainly focused on neurogenesis. The relevance of other brain populations such as microglia has been highlighted, indicating that these tissue-resident brain macrophages are key players in brain homeostasis, development, and diseases (Derecki et al., 2014; Salter and Stevens, 2017). Microglia incorporation into 3D models has not been achieved previously. In this study, we have successfully introduced microglia into our 3D brain spheroids, and demonstrated they alter the response to viral infection. Two techniques were compared to establish the protocol: in the first approach, human microglia immortalized cells were added on top of the NPCs before the formation of the spheres, and then differentiated in 3D over 8 weeks (Supplementary Table S1). We believe that the nature of the microglia used (immortalized by SV40) made this initial protocol impossible due to the constant proliferation leading to a high microglia population. Immortalized cell lines tend to be used as a high-throughput model for experimentation as they are homogeneous in culture. The human microglia immortalized with SV-40 used in our study exhibits biological characteristics such as morphological, phenotypical, and functional properties similar to those documented in primary human microglia (Patel et al., 2016; Zhu et al., 2016; Sousa et al., 2017). Also, immortalized microglia demonstrate appropriate migratory and phagocytic activity, and are capable of eliciting the pro-inflammatory cytokine response, which is characteristic of human microglia (Garcia-Mesa et al., 2017). When adding microglia cells to NPCs (Supplementary Figure Supplementary Table S1, cells added during the aggregation stage with the NPCs), around 50% of the spheres led to overgrowth of microglia and some neuronal cell death (data not shown). The use of iPSC-derived microglia, however, could solve this problem in the future (Muffat et al., 2016). In order to avoid microglial proliferation, microglia cells were added after BS differentiation in the second regimen, after 7 weeks differentiation, and kept for a maximum of 1 week in co-culture (Figure 1A). Microglia were then identified by using different markers such as TMEM119, Mertk, Axl, and IBA1, showing their attachment and growth in the μBS (Figures 1B,C).

The pro-inflammatory stimulus LPS from Gram-negative bacteria as used here and Gram-positive lipoteichoic acid as shown earlier (Kinsner et al., 2005, 2006; Boveri et al., 2006) activate microglia in infections. LPS was used to challenge the μBS system and to compare its effects with both BS and microglia cultures. In response to LPS, microglia produce large quantities of proinflammatory cytokines such as TNF-α, IL-1β, and IL-6. This is normally followed by the production of anti-inflammatory cytokine IL-10 (de Waal Malefyt et al., 1991; Randow et al., 1995). Microglia cultures showed no statistically significant changes neither in pro-inflammatory marker RNA levels (*IL-6* and *IL-1β*) or in the anti-inflammatory marker (*IL-10*). This could be due to the need of other CNS cell types such as astrocytes for the release of these specific cytokines. However, *TNF-α* and *CCL2* were strongly upregulated 24 h after LPS treatment (Figure 2A) which is expected since microglia is the major producer of *TNFα* and *CCL2* is a gene that encodes a small cytokine that recruits monocytes between other inflammatory cells (such as memory T cells and dendritic cells) into the inflammation area. Microglia exposed to LPS showed higher expression (lower Ct values) of most of the genes studied compared to μBS and BS (data no shown). This indicates that microglia express higher levels of these genes, or that in the presence of other cell types in the μBS, these dilute the expression levels of these genes as other cells do not express them (but expression is normalized to housekeeping genes expressed by all cell types). Pro-inflammatory cytokine-encoding genes *IL-6* and *IL-1β* showed a clear increase in expression (Figure 2A) in μBS. Interestingly, the anti-inflammatory marker *IL-10* was also upregulated after LPS treatment in μBS. Cytokines are released very rapidly after inflammatory stimuli and are regulated within a few hours, this could explain the observed co-expression of both anti- and pro-inflammatory genes. The TNF-α gene was not modified significantly after LPS exposure in μBS, BS, and microglia at the early time points. However, microglia exposed to LPS during 24 h LPS exposure showed a high up-regulation. BS and microglia cultures *per se* did not show statistically significant responses to *IL6, IL10,* and *IL1b* at the mRNA level, however, μBS were able to elicit a response which otherwise was not induced in BS after LPS treatment. This indicates that the interaction between microglia and CNS cells (such as neurons, astrocytes and oligodendrocytes) are required for the a specific inflammatory response. This study demonstrates the importance of including microglial populations in *in vitro* models to better predict human pathogenicity.

Flavivirus (DENV-1, ZIKV-BR, and ZIKV-UG) infection induced different responses depending of the model used (microglia, BS or μBS). Flavivirus infection resulted in produced a higher number of RNA virus copies in microglia cells (Figure 4A). Moreover, these viruses were also able to replicate in BS, which is rarely observed *in vitro*. There was an initial increase in viral replication when microglia were added to BS (Figure 4A), however, levels of RNA copies/mL were similar between BS and μBS at 72 h. Microglia showed no changes in viability after virus infection (Figure 4C). ZIKV-BR infection led to higher replication compared to ZIKV-UG and DENV-1 independent of the model, which could indicate that this flavivirus strain is more aggressive than the others (Figure 4A). Moreover, ZIKV-BR infection showed a higher replication rate in microglia and μBS than in BS (Figure 4A) indicating that microglia may somehow influence the replication rate.

Flavivirus infection in μBS and BS showed differences in the expression profile of the genes studied. After infection of μBS by ZIKV-BR and ZIKV-UG, upregulation of *TNF-α,* IL6, IL1b, and *CCL2* was observed when compared with BS (Figure 4D). Moreover, DENV-1 infection led to the upregulation of *CCL2* and *IL-1*β in the presence of microglia (μBS) and upregulation of *TNF-*α and *IL-6* in microglia alone. The μBS model presented a stronger upregulation of all cytokines studied after ZIKV infection, indicating that the interaction between BS and microglia produce more drastic changes in gene expression, which is more relevant to study the physiological response to infection. Moreover, ZIKV-BR infection led to G2/M arrest in μBS when compared to ZIKV-UG and DENV-1 (Figure 4B). Similar results have demonstrated that ZIKV-BR infection deregulates the cell cycle, resulting in attenuated hNPC growth (Dang et al., 2016; Garcez et al., 2016; Tang et al., 2016; Ghouzzi et al., 2017). Furthermore, phagocytosis of apoptotic cells by microglia cells has been described as a general mechanism to clear these cells from tissues (Arandjelovic and Ravichandran, 2015). Duque and Descoteaux described that beside the secretion of cytokines, macrophages can increase their phagocytotic process, contributing to the defense against pathogens (Arango Duque and Descoteaux, 2014).

Our findings suggest that the microglia-driven inflammatory response to ZIKV infection may be exacerbated by endogenous signaling molecules such as viral proteins that can contribute to the pathological damage of neurons. Based on these findings we hypothesize that the activated microglia in μBS play a potential role in enhancing the expression of pro-inflammatory cytokines such as interleukin (IL)-1β, IL-6, and TNF-α in response to ZIKV-BR infection.

In conclusion, the incorporation of human microglia in BS produced an inflammatory response following LPS or flavivirus exposure. This has not been studied previously in a 3D *in vitro* brain model which includes a population of microglial cells. Both exposures produced changes in cell cycle and Annexin V analysis in presence of microglia, suggesting that the microglia-driven inflammatory response induces cytotoxicity leading to a decrease in cell viability. In the future, microglia derived from iPSC-derived microglia may represent an improvement of this model to limit proliferation and extend the co-culture period. As microglia occupy a central position in the defense and maintenance of the CNS, a 3D *in vitro* model including microglia could be fundamental for studies of the brain. Moreover, various anti-inflammatory drugs have been identified in treating microglia-mediated neuroinflammation in the CNS (Baby et al., 2014). The proposed model could serve as a potential tool for the screening of therapeutic targets in neurological disorders and recovery from brain injury. In conclusion, our findings suggest that the μBS model has potential applications as a physiologically relevant model to study infectious disease, host-pathogen interactions, and neuro-inflammation.

## Author Contributions

CA performed the cytokine experiments, virus replication experiments, and live and dead assays. LG contributed to experimental design and the writing of the manuscript in the supervision of CA. SK performed the immunohistochemistry experiments. MC and SO-D performed the BrainSphere cultures. HH and TH assisted with the coordination of the experiments and experimental design and contributed to the discussion. DP designed and coordinated experiments, performed the microglia incorporation, infection, and immunohistochemistry experiments, and wrote the manuscript.

## Conflict of Interest Statement

DP, HH, and TH are named inventors of the Johns Hopkins pending patent application “Compositions and Methods for Neuralgenesis” and benefit from license fees. TH has recently created Organome LLC to make the BrainSpheres commercially available. The remaining authors declare that the research was conducted in the absence of any commercial or financial relationships that could be construed as a potential conflict of interest.

## Supplementary Material

**FIGURE S1 |** Shows the alternative protocol performed to incorporate microglia into BS 3D cultures. When NPCs reached 95% confluence, 1 × 10^6^ microglia were added to the flask. After 24 h, the co-culture was detached mechanically with a cell scraper (Sarstedt, Newton, NC, United States, 2-position, Blade 25, 83.1830), re-pipetted for disaggregation, and counted using the Countess Automated Cell Counter (Invitrogen, Carlsbad, CA, United States). 2 × 10^6^ cells per well were then plated in non-treated 6-well plates and cultured as BS for 8 weeks. The density was chosen to have approximately 10% microglia cells. **(A)** Diagrammatic representation for the alternative protocol. **(B)** Immunohistochemistry showing microglia marker (IBA1) and neuronal marker (NF200). **(C)** Immunohistopathology characterization of the microglia incorporation for this method of μBS generation, markers are mentioned in the main text.
