# Supplementary figures and images for: Microglia Increase Inflammatory Responses in iPSC-Derived Human BrainSpheres
Source: Front Microbiol. 2018 Dec 4;9:2766. doi: 10.3389/fmicb.2018.02766 (PMC6296317; doi:10.3389/fmicb.2018.02766)

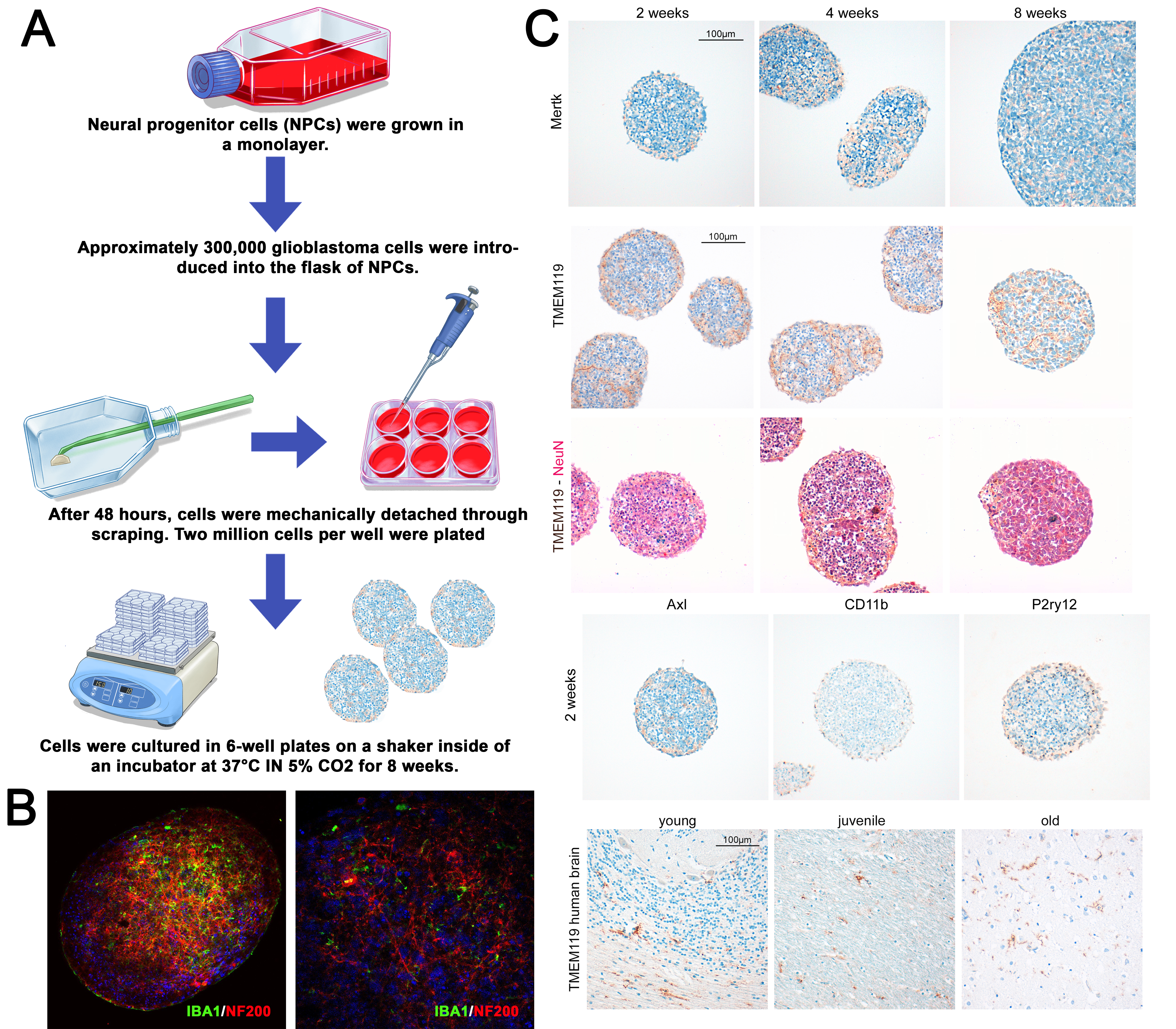

Supplement: Supplementary file 1 [file Image_1.JPEG]
